# A study on the correlation between frailty, sleep quality, and cognitive function in low-income older adults living in the urban–rural fringe of China

**DOI:** 10.3389/fpubh.2026.1834831

**Published:** 2026-05-25

**Authors:** Chicheng Huang, Zijian Zhou, Jingman Yu, Xin Ma, Zhikai Yu, Changming Wang

**Affiliations:** 1 School of Philosophy, Nanjing University, Nanjing, China; 2 School of Mental Health and Psychology, North China University of Science and Technology, Tangshan, China; 3 Ningbo Institute, Dalian University of Technology, Ningbo, China

**Keywords:** cognitive function, daily life activity ability, frailty, low income older adults, mediation analysis, sleep quality, urban–rural fringe

## Abstract

**Objective:**

This cross-sectional study aims to explore the independent and combined effects of physical frailty and sleep quality on cognitive function in low-income older adults in the urban–rural fringe of China, and to examine whether daily activities (ADL) play a mediating role between frailty and cognitive function.

**Methods:**

A combination of convenience sampling and stratified sampling was used to recruit 198 people over 55 and above from a community in the urban–rural fringe of Keerqin District, Tongliao City, Inner Mongolia. Cognitive function was assessed using the Montreal Cognitive Assessment Beijing version (MoCA BJ), frailty was assessed using the FRAIL scale, sleep quality was measured using the Pittsburgh Sleep Quality Index (PSQI), depressive symptoms were assessed using the 15-item version of the Geriatric Depression Scale (GDS-15), and daily functioning was assessed using the ADL scale. Covariates included age, sex, years of education, smoking status, alcohol consumption, and number of chronic diseases. The statistical methods include hierarchical multiple linear regression and Bootstrap mediation analysis (resampling 2,000 times).

**Results:**

48.5% of participants in the sample had cognitive impairment (MoCA<18 points), and 65.2% reported poor sleep quality (PSQI ≥ 5 points). One-way ANOVA showed significant differences in MoCA scores among the three groups of frailty grades (*F* = 7.26, *p* < 0.001, *η*^2^ = 0.069), with the frailty group scoring significantly lower than the robust group (Bonferroni adjusted *p* = 0.001). Hierarchical regression analysis (Model 4, R^2^ = 0.254) showed that the PSQI total score (*B* = −0.78, *p* = 0.016) and ADL score (*B* = 0.85, *p* = 0.010) were independent predictors of MoCA, while the health and lifestyle covariates (smoking, alcohol, chronic disease count) were non-significant. Bootstrap mediation analysis showed that ADL function exhibited a significant mediation effect in the frailty–cognition association (indirect effect = −0.42, 95% CI [−0.80, −0.14]), accounting for 45.4% of the total effect; however, after controlling for lifestyle and comorbidity covariates, this mediation effect was not statistically significant (indirect effect = −0.19, 95% CI [−0.40, 0.03], *p* = 0.092). The sleep–depression–cognition pathway did not reach statistical significance.

**Conclusion:**

Frailty and sleep quality (especially sleep latency) are independently correlated with cognitive function. Daily functional impairment shows a significant mediation trend in the frailty–cognition association, and this effect only reaches marginal significance after controlling for chronic disease burden and lifestyle factors. This suggests that comprehensive interventions addressing multimorbidity may be more fundamental than simple daily functional rehabilitation. Therefore, on the basis of systematic management of multiple chronic diseases, combining interventions aimed at improving sleep difficulties and maintaining physical functional independence can provide practical entry points for promoting cognitive health in this vulnerable group.

## 1 Introduction

The cognitive decline of the older adults is a major global public health challenge. It is predicted that the number of dementia patients in China will increase to 45.54 million by 2050, and the related care costs will exceed 3.2 trillion yuan by 2030 (1, 2). The risk of cognitive impairment among individuals with low socioeconomic status is about 4.4 times higher than that of individuals with high socioeconomic status (3). Identifying modifiable risk factors and underlying mechanisms of cognitive decline in low-income older populations is of great practical significance.

Frailty and sleep disorders are two highly prevalent factors in low-income older populations that are closely related to cognitive aging mechanisms. Frailty is a clinical syndrome characterized by a decrease in physiological reserve capacity, which can accelerate cognitive decline through multiple pathways such as chronic inflammation, reduced cerebral perfusion, and decreased functional capacity (4). Sleep disorders disrupt neural homeostasis through mechanisms such as impairing hippocampal synaptic plasticity, hindering the clearance of beta amyloid protein by the brain lymphatic system, and activating neuroinflammation (upregulation of IL-6 and TNF-*α*) (5–7). Low socioeconomic status can amplify the risk of sleep disorders through noise, chronic stress, and poor living conditions (8), further accelerating cognitive aging.

Although previous studies have separately confirmed the independent association between the above factors and cognitive function, research on the combined effects and mechanisms of frailty and sleep quality in low-income older populations in the urban–rural fringe of China is still very limited. The urban–rural fringe is a special field born from China’s rapid urbanization process. Residents in this area are simultaneously under the dual pressure of environmental disadvantages (noise, pollution) brought about by urban expansion and insufficient supply of public services at the rural level (9). They also face obvious “marginalization” difficulties at the social and psychological level (10).

Among the potential mechanisms by which frailty affects cognition, impaired daily functioning is a candidate mediating pathway that deserves special attention. Weakness leads to decreased physical activity and reduced independence of daily functioning, and ADL impairment has been shown to be independently associated with cognitive decline (11). If ADL is indeed a mediating variable in the frailty cognitive association, interventions that maintain functional independence are expected to produce cognitive protective benefits in situations where frailty itself is difficult to completely reverse.

Despite growing evidence on frailty and sleep quality as independent risk factors for cognitive decline, their combined effects and underlying mechanisms in low-income peri-urban older populations in China remain largely unexplored. This gap is particularly important given the amplified vulnerability of this group to environmental stressors and limited healthcare access.

**Figure 1:**
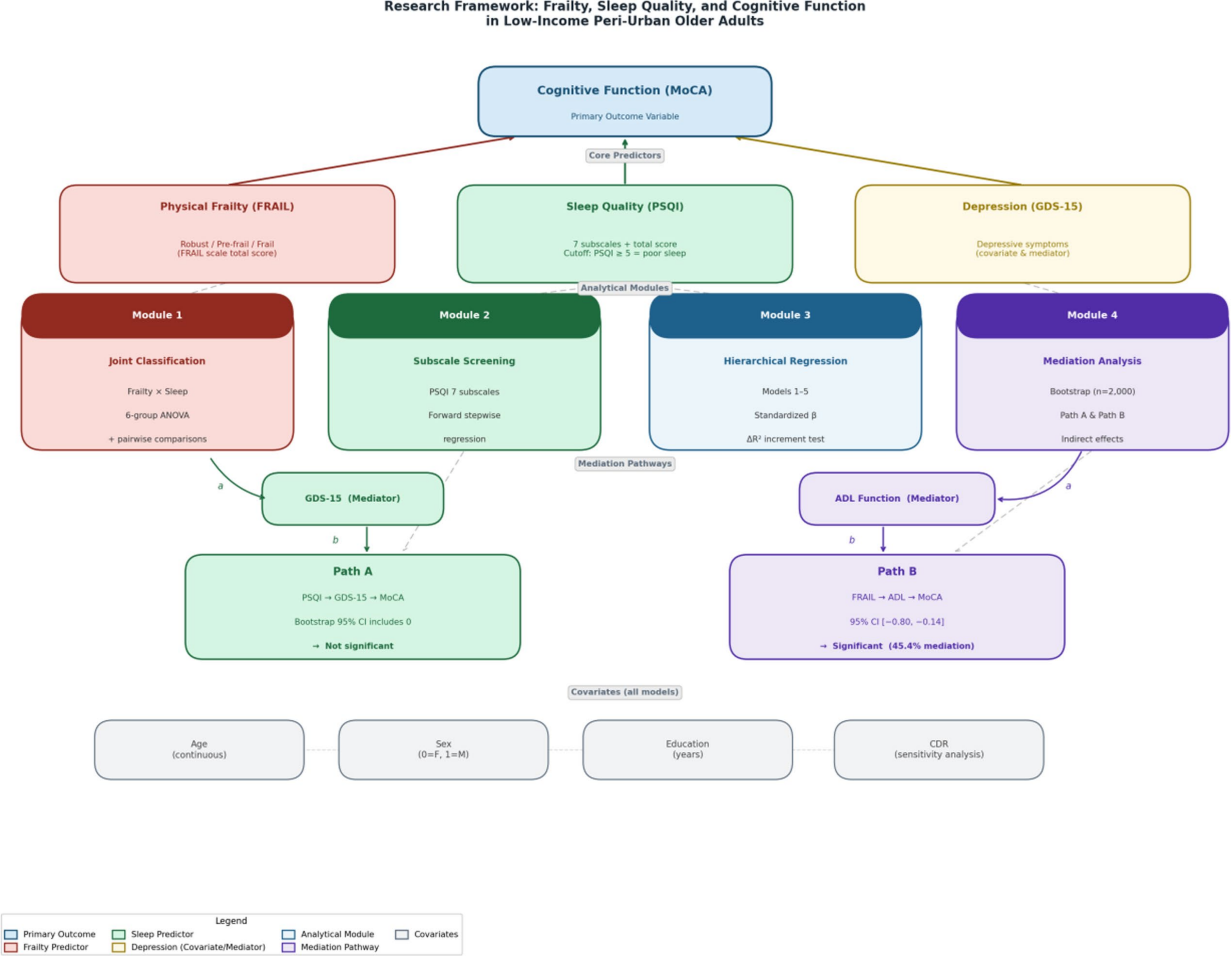
Research framework. The diagram illustrates the conceptual structure of the study, showing the primary outcome (MoCA), core predictors (frailty, sleep quality, depression), four analytical modules, hypothesized mediation pathways (Path A: PSQI→GDS-15 → MoCA; Path B: FRAIL→ADL → MoCA), and covariates included in all models.

Based on the above background, this study selected 198 low-income older adults people from a community in the urban–rural fringe of northern China as research subjects, aiming to: (1) clarify the independent effects of frailty and sleep quality on cognitive function; (2) examine whether there is a combined effect of frailty and sleep quality on cognitive function; (3) test whether ADL function mediates the association between frailty and cognitive function, and whether depressive symptoms mediate the sleep-cognition association (see Figure 1).

## 2 Research methods

### 2.1 Research object and venue

This cross-sectional study was conducted from January to February 2025 in a mixed urban–rural community in Keerqin District, Tongliao City, Inner Mongolia Autonomous Region. A combination of stratified and convenience sampling was used to recruit 198 people over 55 and above living in the community. Participants have limited formal retirement income and mostly reside in urban–rural fringe areas or urban villages. The median monthly income per capita of the recruited community is 1.5 times lower than the local minimum living allowance standard (864 yuan), which is consistent with the low-income characteristics. According to the 2024 Tongliao municipal Minimum Living Allowance standards, ‘low income’ is operationally defined as urban household per capita monthly income below 864 yuan or rural household per capita annual net income below 7,668 yuan. Age was used as the stratification variable for sample planning. The target community was purposefully selected as it is situated at a typical peri-urban expansion boundary, where residents are predominantly former agricultural workers lacking formal pension benefits. To minimize selection bias inherent in convenience sampling, the following measures were implemented: (a) diversified recruitment through both community center-based and door-to-door approaches via community grid workers, ensuring inclusion of frail, homebound older adults; (b) standardized face-to-face assessment by uniformly trained investigators; and (c) strict adherence to pre-defined inclusion and exclusion criteria.

**Figure 2:**
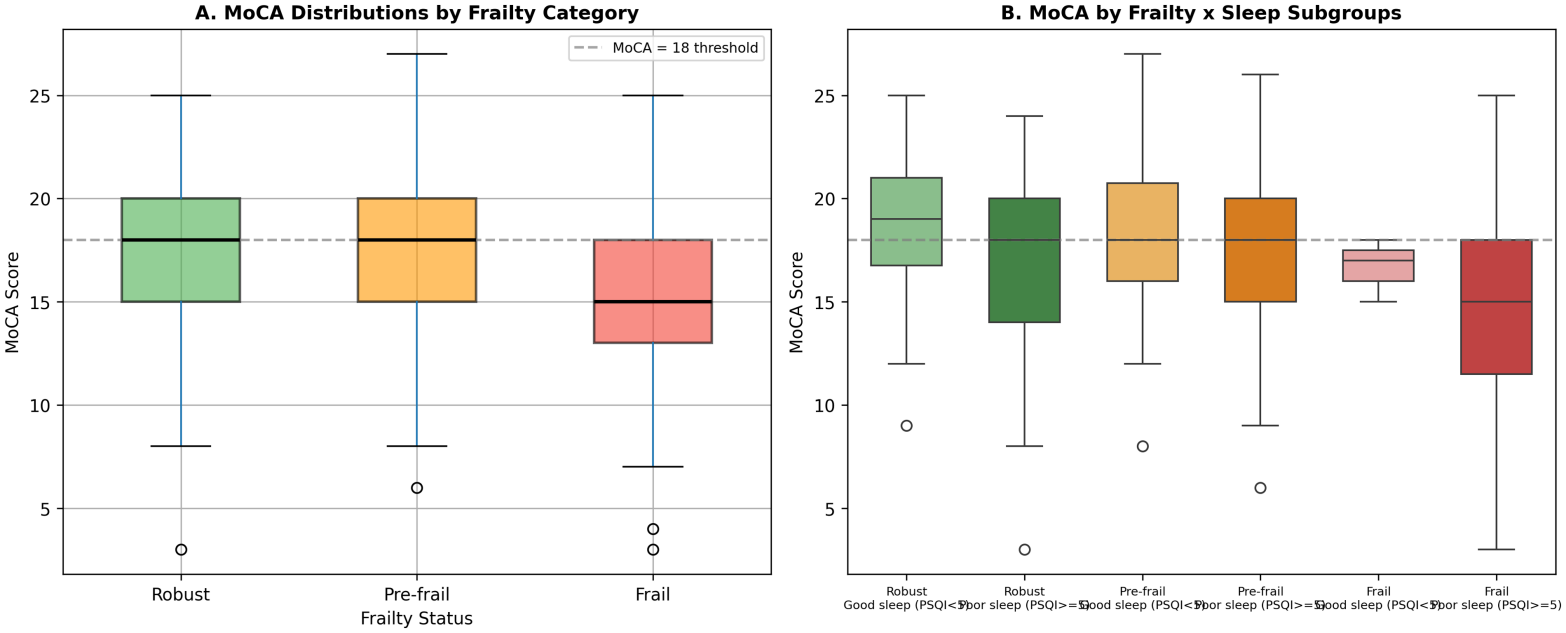
Frailty status, sleep quality, and MoCA scores. **(A)** MoCA distributions by frailty category. **(B)** MoCA by six joint frailty × sleep subgroups. Dashed line = MoCA 18 threshold.

*Inclusion criteria*: age ≥ 55 years old; Have basic self-care ability (ADL score<22); Ability to understand and complete structured scale assessments; Sign the informed consent form and voluntarily participate in the study. Exclusion criteria: Severe mental or neurological disorders, sensory or motor impairments that affect assessment completion, current use of drugs known to affect cognitive function (such as sedatives, antidepressants, antipsychotics), and recent hospitalization history for major illnesses. This study follows the latest version of the Helsinki Declaration and has been approved by the Medical Ethics Committee of North China University of Technology (Approval Number: 2024085). Written informed consent was obtained from all participants prior to data collection. Consent forms were signed in the presence of trained assessors and retained in the study files (see Figure 2).

Perform prior statistical power analysis using G* Power 3.1 software, with a test power of 0.95, significance level *α* = 0.05, moderate effect size *f*^2^ = 0.15, and a maximum of 7 predictor variables. The minimum sample size required for calculation is 146 cases. This study ultimately included 198 cases, with sufficient statistical power. A total of 215 older residents were initially approached for enrollment, of whom 12 declined participation and 5 were excluded based on exclusion criteria, resulting in a final analytic sample of 198 (response rate: 92.1%). Missing data were minimal (<2% for all variables). Listwise deletion was used in all analyses, yielding analytic sample sizes ranging from 194 to 198 depending on the specific model.

### 2.2 Measuring tools

#### 2.2.1 Cognitive function

The Montreal Cognitive Assessment Scale Beijing version was used to assess cognitive function, covering 8 areas including visual space/executive function, naming, memory, attention, language, abstraction, delayed recall, and orientation, with a total score of 30 points. The MoCA score of <18 is used as the cognitive impairment standard (adjusted for years of education according to the standard scoring criteria). According to the reference criteria adopted in this study, the Beijing version of the MoCA (MoCA-BJ) demonstrated an internal consistency of *α* = 0.73 and a test–retest reliability of ICC = 0.87 (12, 13).

#### 2.2.2 Physical frailty

The FRAIL scale was used to assess physical frailty, which includes five items: fatigue, endurance (ability to climb a staircase independently), walking ability (ability to walk a block), complications (≥5 chronic diseases), and weight loss (weight loss >5% in the past year), with a total score of 0–5 points. Strong: 0 points; Early stage of frailty: 1 point; Weakness: ≥2 points. According to the reference criteria adopted in this study, Internal consistency *α* = 0.826; inter-rater Kappa = 0.892 (14, 15).

#### 2.2.3 Sleep quality

The Pittsburgh Sleep Quality Index was used to evaluate sleep quality over the past month, which includes seven components: subjective sleep quality, latency to sleep, sleep time, sleep efficiency, sleep disorders, hypnotic drug use, and daytime dysfunction. The global total score is 0–21 points. The cutoff value for poor sleep quality is a total score of ≥5. According to the reference criteria adopted in this study, the Chinese version of the PSQI has demonstrated an internal consistency of *α* = 0.734 and a test–retest reliability of *r* = 0.994 (16, 17).

#### 2.2.4 Depressive symptoms

The 15-item version of the Geriatric Depression Scale was used to assess depressive symptoms, with a total score of 0–15 points. A score of ≥5 indicates the presence of clinically significant depressive symptoms. According to the reference criteria adopted in this study, the GDS-15 demonstrated an internal consistency (Cronbach’s alpha) of 0.82 (18, 19).

#### 2.2.5 Daily functions and covariates

Activities of Daily Living (ADL) were assessed using the ADL scale, with higher scores indicating stronger functional independence. The covariates include: years of education (cognitive reserve proxy, included in all models), age (continuous), sex (0 = female, 1 = male), smoking status (0 = no, 1 = yes), alcohol consumption (0 = no, 1 = yes), and number of chronic diseases (continuous). All continuous predictors were z-score standardized prior to entry. According to the reference criteria adopted in this study, Convergent validity Kappa with Barthel Index = 0.875 (20, 21).

#### 2.2.6 Cognitive impairment screening

The Chinese version of the Clinical Dementia Rating scale (CDR) was administered to assess cognitive functional status and dementia severity. CDR is a semi-structured interview instrument assessing six domains: memory, orientation, judgment and problem solving, community affairs, home and hobbies, and personal care. Trained clinicians conducted independent interviews with both the participant and an informed collateral source (family member or primary caregiver). Each domain was rated on a 5-point scale (0, 0.5, 1, 2, 3), with the global CDR score determined using the standard algorithm with memory as the primary category. A CDR score of 0 indicates normal cognition, 0.5 indicates questionable cognitive impairment (MCI), and ≥1 indicates probable dementia. In the sensitivity analysis, participants with CDR ≥ 1 were excluded to test the robustness of the main findings in a non-demented subsample, thereby mitigating the risk of reverse causality (i.e., dementia itself causing sleep disturbance and functional decline).”

### 2.3 Statistical analysis

**Figure 3:**
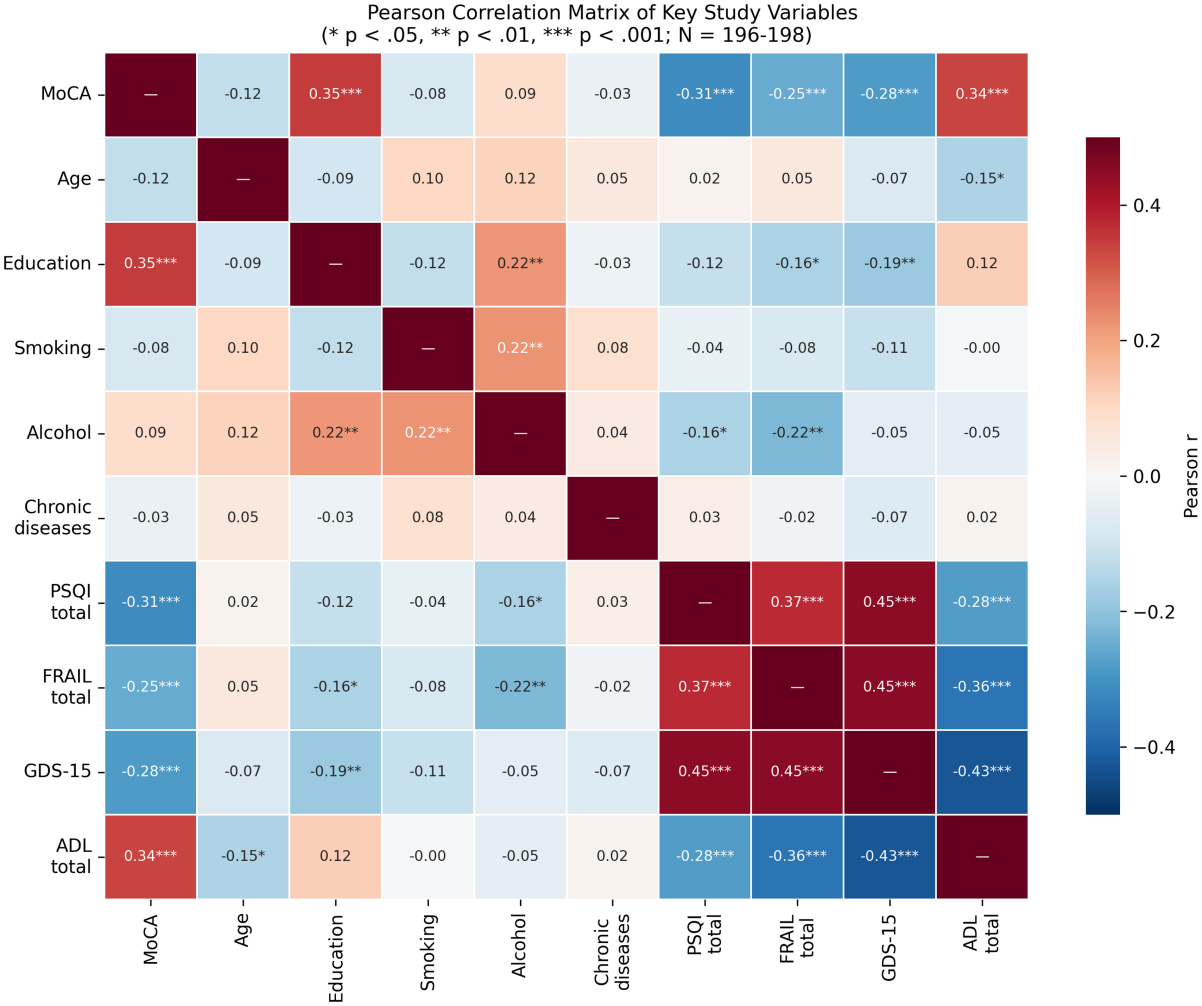
Pearson correlation matrix. Color intensity reflects magnitude; blue = positive, red = negative associations.

All statistical analyses were conducted using Python 3.12 (NumPy, SciPy). Continuous variables are described as mean ± standard deviation (M ± SD), while categorical variables are described as frequency and percentage (n, %) (Table 1). The Pearson correlation coefficients between the main continuous variables are shown in Figure 3.

**Table 1:** Descriptive statistics.

Variable	M (SD) or n (%)	Range	n
Continuous variables			
Age (years)	70.17 ± 6.23	55–87	198
Education (years)	4.43 ± 3.02	0–12	197
Number of chronic diseases	0.91 ± 0.71	0–4	196
MoCA score	17.08 ± 4.34	3–27	198
PSQI total score	7.34 ± 5.08	0–20	198
FRAIL total score	0.82 ± 1.08	0–5	198
GDS-15 total score	2.85 ± 2.62	0–11	198
ADL total score	12.77 ± 2.26	1–15	197
Categorical variables			
Sex: male	88 (44.4%)		198
Smoking (yes)	44 (22.2%)		198
Alcohol consumption (yes)	57 (28.8%)		198
Frailty: robust (score = 0)	101 (51.0%)		198
Frailty: Pre-frail (score = 1)	60 (30.3%)		198
Frailty: frail (score ≥ 2)	37 (18.7%)		198
Poor sleep (PSQI ≥ 5)	129 (65.2%)		198
Cognitive impairment (MoCA < 18)	96 (48.5%)		198
Depression symptoms (GDS ≥ 5)	41 (20.7%)		198

MoCA, Montreal cognitive assessment; PSQI, Pittsburgh sleep quality index; GDS-15, geriatric depression scale-15; FRAIL, fatigue, resistance, ambulation, illness, loss of weight; ADL, activities of daily living. Valid cases are *n* = 198 for all variables except education (*n* = 197), ADL (*n* = 197), and chronic diseases (*n* = 196).

Based on controlling for age, gender, and years of education, this study used the forward stepwise regression method to analyze the independent effects of the seven components of the PSQI on the MoCA (inclusion criterion: *p* < 0.05). The choice of this method has a solid statistical basis and necessity: given the high internal correlation among the PSQI subscales, if all components were included in the model simultaneously, it would inevitably cause serious multicollinearity, thereby distorting the analysis results. Therefore, using the forward stepwise regression method is the optimal strategy to avoid multicollinearity and accurately extract independent predictors.

Five nested models were constructed using hierarchical multiple linear regression to evaluate the independent predictive factors of MoCA. Model 1 includes age, sex, smoking status, alcohol consumption, and number of chronic diseases as baseline demographic and health covariates; Model 2 adds years of education, FRAIL total score, and PSQI total score; Model 3 adds the GDS-15 total score; Model 4 adds the ADL total score; Model 5 adds the FRAIL × PSQI interaction term. All continuous predictor variables were z-score standardized before entry. The goodness of fit of the model is evaluated using *F*-tests for *R*^2^, corrected *R*^2^, and Δ*R*^2^.

Bootstrap mediation analysis (resampling 2000 times) examined two pathways: pathway A, whether depressive symptoms (GDS-15) mediate the association between sleep quality (PSQI) and cognitive function (MoCA); Path B: Does ADL function mediate the association between frailty (FRAIL) and cognitive function. Both models control for age, sex, years of education, smoking status, alcohol consumption, and number of chronic diseases. When the 95% Bootstrap confidence interval of the indirect effect (a × b) does not include 0, it is judged that the mediating effect is significant.

Sensitivity analysis includes: (1) re running the main model after excluding participants with CDR ≥ 1; (2) Stratified analysis by gender; (3) Stratified analysis by age (<70 years vs. ≥70 years). All tests are two-sided tests, with a significance level set at *α* = 0.05.

## 3 Results

### 3.1 Sample characteristics

A total of 198 participants were ultimately included, with an average age of 70.2 ± 6.2 years, 55.6% of whom were female, and an average length of education of 4.4 ± 3.0 years. The average score of MoCA is 17.1 ± 4.3 points, and 96 people (48.5%) have cognitive impairment (MoCA<18 points). Weakness grading: 101 people were strong (51.0%), 60 people were in the early stage of weakness (30.3%), and 37 people were weak (18.7%). 129 people (65.2%) had poor sleep quality (PSQI ≥ 5), with an average PSQI score of 7.3 ± 5.1. 41 people (20.7%) had a GDS-15 score of ≥5 (positive for depressive symptoms). The mean number of chronic diseases was 0.91 ± 0.71 (range 0–4). 44 participants (22.2%) were current smokers, and 57 (28.8%) reported alcohol consumption. See Table 1 for details.

### 3.2 Correlation analysis between variables

Figure 3 presents the Pearson correlation matrix of the 10 main study variables, MoCA score was significantly negatively correlated with PSQI total score (*r* = −0.31, *p* < 0.001), FRAIL total score (*r* = −0.25, *p* < 0.001), and GDS-15 (*r* = −0.28, *p* < 0.001), and significantly positively correlated with years of education (*r* = 0.35, *p* < 0.001) and ADL total score (*r* = 0.34, *p* < 0.001). PSQI was moderately correlated with both GDS-15 (*r* = 0.45, *p* < 0.001) and FRAIL (*r* = 0.37, *p* < 0.001), supporting the inclusion of depression and frailty as covariates. Among the health behavior variables, alcohol consumption was significantly negatively correlated with FRAIL (r = −0.22, *p* < 0.01) and PSQI (*r* = −0.16, *p* < 0.05), while smoking and chronic disease count showed no significant correlations with MoCA.

### 3.3 PSQI component analysis

The forward stepwise regression results showed that after controlling for age, gender, and years of education, only the “sleep onset latency” significantly independently predicted MoCA scores among the seven PSQI components (*B* = −1.03, SE = 0.23, *t* = −4.58, *p* < 0.001). The final model explained 21.8% of MoCA variance (adjusted *R*^2^ = 0.202), an increase of 8.5 percentage points compared to the baseline demographic model. The remaining six components (subjective sleep quality, sleep time, sleep efficiency, sleep disorders, hypnotic drug use, daytime dysfunction) did not reach statistical significance during the gradual screening process (see Table 2).

**Table 2:** Hierarchical multiple linear regression analysis of factors associated with cognitive function MoCA scores.

Variable	Model 1		Model 2		Model 3		Model4		Model 5	
	B	SE(95% CI)	B	SE(95% CI)	B	SE(95% CI)	B	SE(95% CI)	B	SE(95% CI)
Intercep	16.99***	0.43 [16.14, 17.84]	17.51***	0.40 [16.72, 18.30]	17.51***	0.40 [16.72, 18.29]	17.31***	0.40 [16.52, 18.10]	17.35***	0.41 [16.53, 18.17]
Age (*z*)	−0.50	0.31 [−1.11, 0.12]	−0.31	0.29 [−0.87, 0.26]	−0.37	0.29 [−0.93, 0.20]	−0.26	0.29 [−0.82, 0.31]	−0.27	0.29 [−0.83, 0.30]
Sex	0.04	0.76 [−1.46, 1.53]	−0.90	0.71 [−2.29, 0.49]	−0.96	0.70 [−2.35, 0.43]	−0.55	0.71 [−1.96, 0.85]	−0.54	0.71 [−1.95, 0.86]
Smoking	−1.27	0.77 [−2.79, 0.24]	−0.69	0.73 [−2.12, 0.74]	−0.83	0.73 [−2.27, 0.60]	−0.81	0.72 [−2.23, 0.61]	−0.84	0.73 [−2.27, 0.59]
Drinking	1.02	0.84 [−0.64, 2.68]	0.26	0.78 [−1.27, 1.79]	0.48	0.79 [−1.07, 2.03]	0.48	0.77 [−1.04, 2.01]	0.51	0.78 [−1.03, 2.04]
Number of chronic diseases (*z*)	−0.09	0.31 [−0.70, 0.52]	0.00	0.28 [−0.56, 0.56]	−0.04	0.28 [−0.59, 0.52]	−0.06	0.28 [−0.61, 0.49]	−0.05	0.28 [−0.60, 0.50]
Education (*z*)			1.29***	0.30 [0.69, 1.88]	1.21***	0.31 [0.60, 1.81]	1.15***	0.30 [0.55, 1.75]	1.16***	0.30 [0.56, 1.76]
PSQI total (*z*)			−1.04***	0.30 [−1.63, −0.44]	−0.86**	0.32 [−1.49, −0.22]	−0.78*	0.32 [−1.41, −0.15]	−0.76*	0.32 [−1.40, −0.13]
FRAIL total (*z*)			−0.55	0.31 [−1.16, 0.06]	−0.37	0.33 [−1.02, 0.28]	−0.19	0.33 [−0.85, 0.46]	−0.14	0.36 [−0.84, 0.57]
GDS-15 (*z*)					−0.55	0.34 [−1.22, 0.13]	−0.26	0.36 [−0.96, 0.44]	−0.25	0.36 [−0.96, 0.45]
ADL total (*z*)							0.85*	0.33 [0.20, 1.49]	0.83*	0.33 [0.18, 1.48]
FRAIL × PSQI (*z*)									−0.12	0.27 [−0.66, 0.42]
*N*	196		195		195		194		194	
*R* ^2^	0.035		0.219		0.229		0.254		0.255	
Adjusted *R*^2^	0.010		0.185		0.192		0.214		0.210	
F for Δ*R*^2^	1.40		6.51***		6.11***		6.24***		5.67***	

*B* = regression coefficient. All continuous predictors were z-score standardized before entry, whereas the outcome variable (MoCA) was retained in its original metric. SE = standard error. Δ*R*^2^ = incremental explained variance (effect size for model comparison) Sex: 0 = female, 1 = male. FRAIL × PSQI = interaction term. *p < 0.05, **p < 0 0.01, ***p < 0.001.

### 3.4 The combined effect of frailty and sleep quality

One-way ANOVA showed significant differences in MoCA scores among the three groups of frailty grades [*F*(2,195) = 7.26, *p* < 0.001, *η*^2^ = 0.069]. After Bonferroni correction, pairwise comparisons showed that the MoCA in the frailty group was significantly lower than that in the strong group (*p* = 0.001) and the pre frailty group (*p* = 0.006), while there was no statistically significant difference between the strong group and the pre frailty group (*p* = 1.00). The average MoCA score in patients with poor sleep quality was significantly lower than that in patients with good sleep [(16.37 ± 4.43 vs. 18.41 ± 3.81); *F*(1,196) = 10.32, *p* = 0.002]. In the six category joint grouping, the frailty/poor sleep group had the lowest mean MoCA score (M = 14.53, SD = 4.71, *n* = 34), while the strong/good sleep group had the highest mean score (M = 18.52, SD = 3.65).

### 3.5 Hierarchical multiple regression analysis

In this study, hierarchical multiple linear regression was conducted to identify independent predictors of MoCA. In the regression analyses, all continuous predictors were z-standardized prior to entry, whereas MoCA was retained in its original scale. Therefore, the reported coefficients are *B* values rather than fully standardized *β* coefficients, and they represent the expected change in raw MoCA score associated with a one-standard-deviation increase in each predictor. Δ*R*^2^ reflects the incremental change in explained variance after adding a new block of variables. Model fit was assessed using *R*^2^, adjusted *R*^2^, and Δ*R*^2^, with effect size metrics consistently reported across analyses.

The baseline covariate model (Model 1: age, sex, smoking, alcohol, chronic disease count) did not reach statistical significance (*R*^2^ = 0.035, *p* = 0.227). After adding years of education, FRAIL total score, and PSQI total score in Model 2, the model showed a significant improvement (Δ*R*^2^ = 0.184, *p* < 0.001), indicating an increase in explained variance. Years of education (*B* = 1.29, *p* < 0.001) and PSQI (*B* = −1.04, *p* = 0.001) were identified as significant predictors, whereas the FRAIL total score approached but did not reach statistical significance (*B* = −0.55, *p* = 0.077). After adding GDS-15 in Model 3, the PSQI coefficient slightly decreased (*B* = −0.86, *p* = 0.009), suggesting a potential partial confounding effect of depressive symptoms; however, GDS-15 itself did not reach statistical significance (*B* = −0.55, *p* = 0.115). In Model 4, after further adding the ADL total score, the model showed a modest but significant improvement (Δ*R*^2^ = 0.025, *p* = 0.010). Both PSQI (*B* = −0.78, *p* = 0.016) and ADL (*B* = 0.85, *p* = 0.010) were significantly associated with MoCA, whereas the FRAIL total score was not statistically significant (*B* = −0.19, *p* = 0.564). Lifestyle and comorbidity-related covariates (smoking, alcohol use, and chronic disease count) were not significantly associated with MoCA across all models. Finally, the addition of the FRAIL × PSQI interaction term in Model 5 did not yield a statistically significant effect (*B* = −0.12, *p* = 0.664), suggesting no evidence of an interaction between frailty and sleep quality in predicting MoCA in the current sample.

### 3.6 Bootstrap intermediary analysis

**Figure 4:**
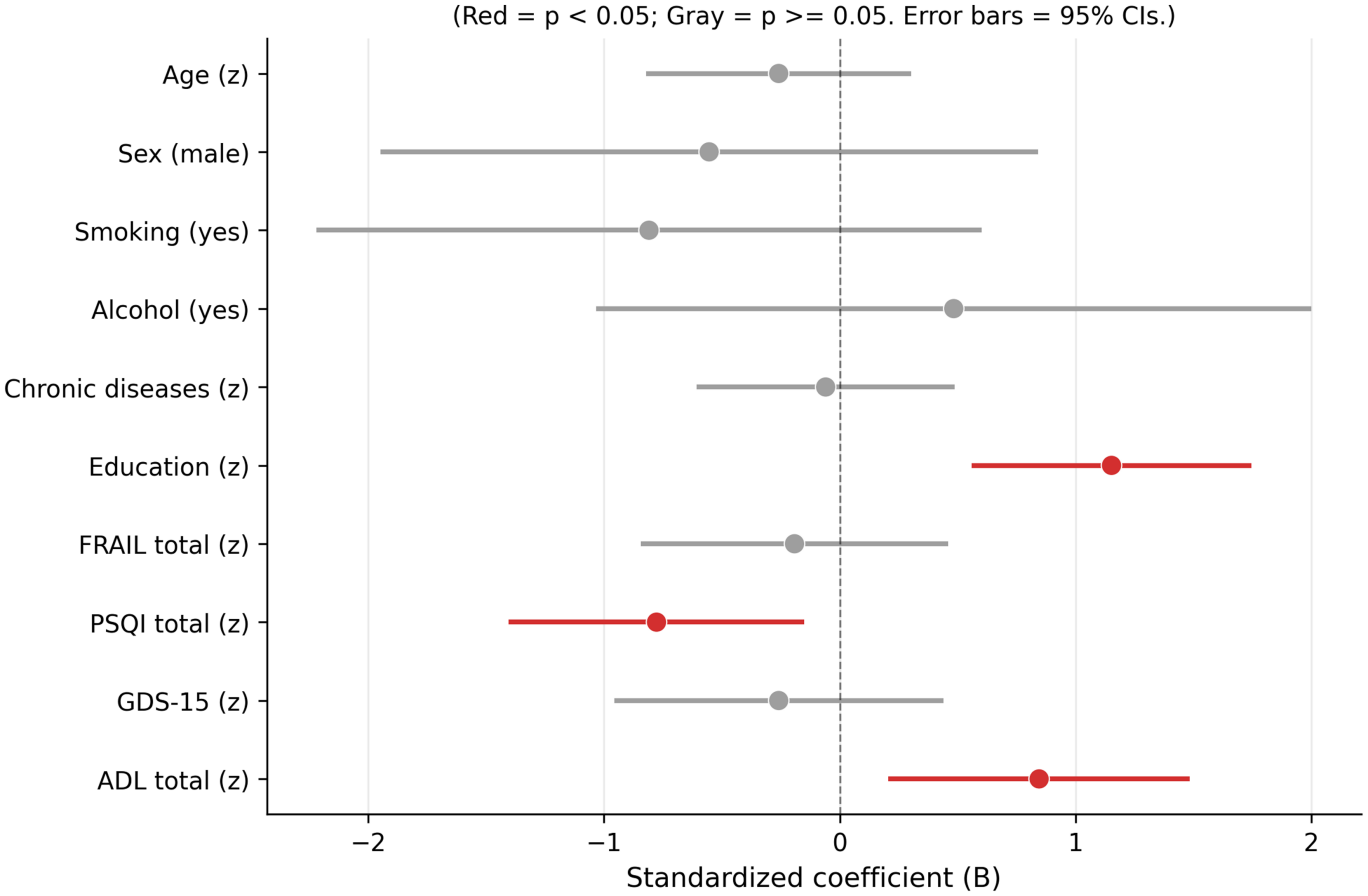
Forest plot of standardized regression coefficients, Model 4. Red = *p* < 0.05; gray = *p* ≥ 0.05. Error bars represent 95% CIs. Model includes age, sex, smoking, alcohol, chronic disease count, education, PSQI, FRAIL, GDS-15, and ADL as predictors.

The results of the intermediary analysis are shown in Table 3. Path A tests the mediating effect of depressive symptoms on the sleep–cognition association: sleep quality was significantly associated with GDS-15 (*a* = 0.33, *p* < 0.001), but after controlling for PSQI and all covariates, GDS-15 did not show a significant effect on MoCA (*b* = −0.55, *p* = 0.121). The indirect effect was −0.18 (95% Bootstrap CI [−0.42, 0.06]), which did not reach statistical significance as the confidence interval included zero. The direct effect of PSQI on MoCA remained significant (*c*′ = −0.86, *p* = 0.015) (see Figure 4).

**Table 3:** Bootstrap mediation results (2,000 resamples).

Path	*a*	*b*	*c*′	Indirect (*a* × *b*)	Bootstrap 95% CI	Significant
Path A: PSQI → GDS-15 → MoCA	0.33***	−0.55	−0.86*	−0.18	[−0.42, 0.06]	No
Path B: FRAIL → ADL → MoCA	−0.22**	0.85*	−0.19	−0.19	[−0.40, 0.03]	Marginal (*p* = 0.092)

Covariates in all models: age (*z*), sex, education (*z*), smoking, alcohol, chronic disease count (*z*). **p < 0.01, ***p < 0.001.

**Figure 5:**
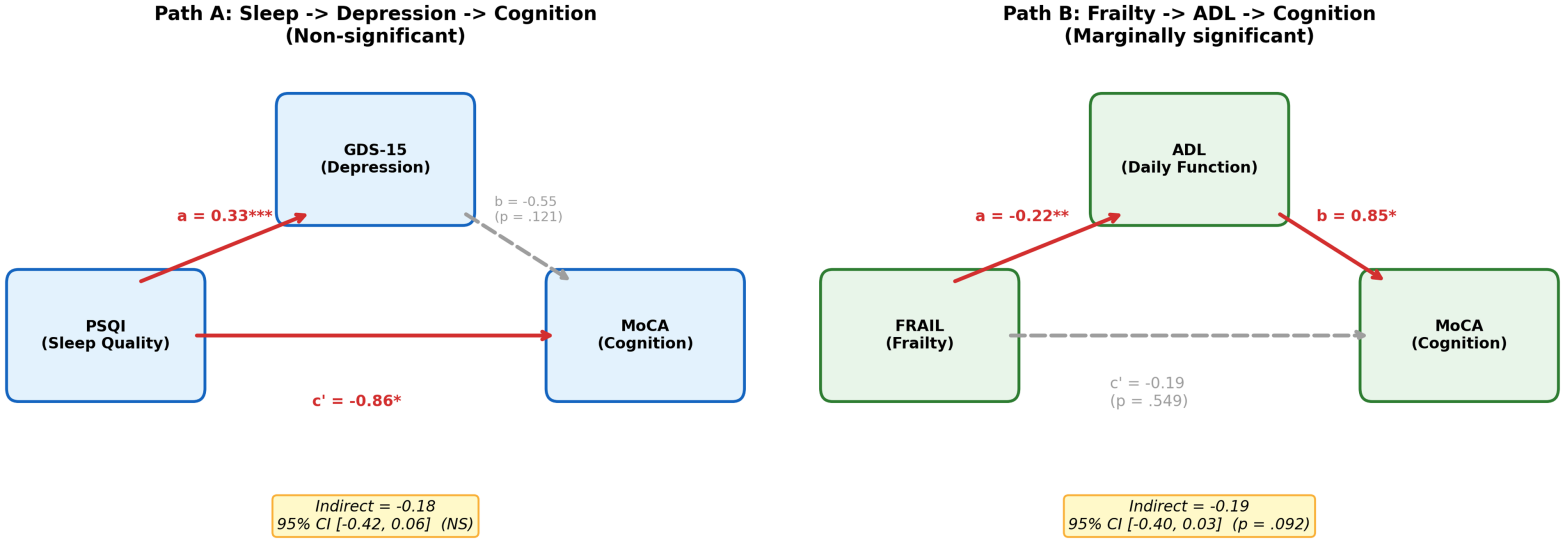
Mediation pathway diagrams (Bootstrap *n* = 2,000). Path A: PSQI to GDS-15 to MoCA (non-significant). Path B: FRAIL to ADL to MoCA (marginally significant, 95% CI includes zero, *p* = 0.092). All models control for age, sex, education, smoking, alcohol, and chronic disease count.

Path B aims to examine the mediating effect of activities of daily living on the association between frailty and cognition. In the base model without additional covariates, ADL showed a strong mediating effect: FRAIL total score was significantly associated with ADL (*a* = −0.39, *p* < 0.001), and ADL had a significant effect on MoCA (*b* = 1.07, *p* < 0.001); the mediating effect was significant (indirect effect = −0.42, 95% CI: [−0.80, −0.14]), accounting for about 45.4% of the total effect. This negative value indicates that increased frailty lowers cognitive scores via ADL impairment. However, after strictly controlling for all covariates such as lifestyle and comorbidities, the effect of this mediating pathway was greatly weakened. Although FRAIL still predicted ADL (*a* = −0.22, *p* = 0.003), and ADL still had a positive effect on MoCA (*b* = 0.85, *p* = 0.023), the indirect effect was reduced to −0.19, and the 95% confidence interval marginally included 0 ([−0.40, 0.03], *p* = 0.092). In both models, the direct effect after controlling for ADL was no longer significant (uncorrected: *c*′ = −0.50, *p* = 0.173; corrected: *c*′ = −0.19, *p* = 0.549) (see Figure 5).

### 3.7 Sensitivity analysis

**Figure 6:**
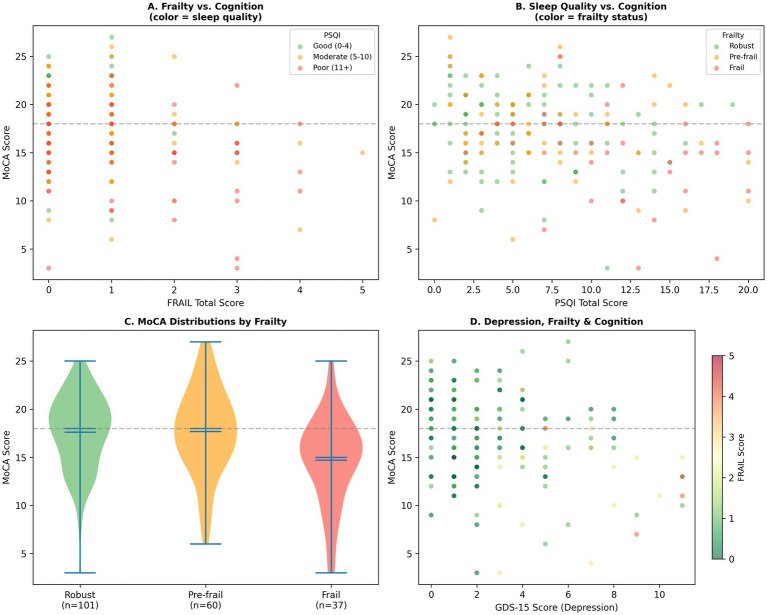
Summary panel. **(A)**: Frailty vs. cognition (color = sleep quality). **(B)**: Sleep quality vs. cognition (color = frailty). **(C)**: MoCA distributions by frailty category. **(D)**: Depression, frailty, and cognition.

After excluding participants with CDR ≥ 1 (*n* = 157), the PSQI coefficient maintained its negative direction but did not reach significance (*B* = −0.55, *p* = 0.121), likely attributable to reduced statistical power from the smaller sample and restricted MoCA variance after removing the most cognitively impaired participants. Stratified by sex, PSQI was a significant predictor in males (*B* = −1.45, *p* = 0.005) but not in females (*B* = −0.59, *p* = 0.161). Stratified by age, PSQI remained significant in the under-70 group (*B* = −1.36, *p* = 0.010) but did not reach significance in the 70-and-above group (*B* = −0.60, *p* = 0.142). The FRAIL coefficient maintained a consistent negative direction in most subsamples but did not reach statistical significance in any, likely due to the additional health baseline covariates and reduced subgroup sample sizes (see Table 4 and Figure 6).

**Table 4:** Sensitivity analyses: standardized regression coefficients across subsamples.

Subsample	*n*	PSQI (*z*) *B* (SE)	*p*	FRAIL (*z*) *B* (SE)	*p*
Exclude CDR ≥ 1	157	−0.55 (0.35)	0.121	−0.02 (0.35)	0.965
Male	86	−1.45 (0.50)	0.005**	−0.50 (0.61)	0.414
Female	108	−0.59 (0.42)	0.161	−0.05 (0.40)	0.903
Age < 70	94	−1.36 (0.52)	0.010*	0.37 (0.49)	0.457
Age ≥ 70	100	−0.60 (0.41)	0.142	−0.35 (0.44)	0.428

All models include the full set of covariates (age, sex, education, smoking, alcohol, chronic disease count, PSQI, FRAIL, GDS-15, ADL). Sex excluded from sex-stratified models. *p < 0.05, **p < 0.01.

## 4 Discussion

This study examined the correlation and mechanism between physical frailty and sleep quality on cognitive function in a sample of low-income older adults communities in the urban–rural fringe of northern China. Three main findings were obtained: firstly, there was a significant correlation between frailty and cognitive function, with the frailty group having an average MoCA score about 4 points lower than the robust group; Secondly, poor sleep quality, especially prolonged sleep latency, independently predicted lower cognitive scores across all regression models; Thirdly, ADL showed a significant mediation effect in the frailty–cognition association, but after controlling for lifestyle and comorbidity covariates, ADL functional impairment showed only a marginally significant mediation trend in the frailty–cognition association, The mediating pathway of sleep–depression–cognition was not statistically supported.

Our finding that ADL mediation was attenuated after controlling for chronic disease burden contrasts with studies in higher-income or healthier community samples, where functional impairment showed stronger and more consistent mediation of the frailty–cognition link (22). We attribute this discrepancy to the high comorbidity burden in our low-income urban–rural fringe sample: when chronic disease count is entered as a covariate, a substantial portion of the frailty–ADL–cognition variance is absorbed by shared disease-related pathways (23), leaving ADL with a more limited independent mediating role. This interpretation aligns with the multimorbidity framework proposing that multiple co-occurring diseases collectively drive functional and cognitive decline through overlapping biological mechanisms, rendering single-pathway mediation analyses less sensitive in high-comorbidity populations (24).

The specificity of the impact of sleep latency on cognitive function has been suggested in previous studies, and this study further confirms it in low-income older populations. Extended latency to sleep is a core manifestation of excessive wakefulness (25), which is associated with reduced slow wave sleep and subsequently affects the clearance of beta amyloid protein by the brain lymphatic system (26). It is worth noting that other sleep dimensions were not significant after controlling for covariates, which may also be partly due to self-reported recall bias - compared to other sleep dimensions, participants with cognitive impairment have more reliable memories of difficulty falling asleep. This robust recall performance for sleep latency is not an isolated finding. This observation is consistent with evidence that sleep initiation difficulties are among the most salient and reliably reported sleep complaints even in individuals with mild-to-moderate cognitive impairment. The subjective experience of prolonged wakefulness at sleep onset is phenomenologically distinctive and temporally bounded, making it less susceptible to retrospective distortion than more diffuse dimensions such as sleep efficiency or nocturnal awakenings. According to existing research, it has been demonstrated that cognitively impaired older adults show preserved complaint accuracy for sleep latency relative to actigraphic measures (27), and it has been shown that NREM sleep disruption—closely related to sleep onset difficulties—is linked to hippocampal atrophy and amyloid accumulation (28), providing a neurobiological basis for the specificity of sleep latency as a cognitive risk indicator in this population.

The depression mediation pathway (Path A) did not reach statistical significance. Two complementary explanations should be considered. First, the marginality of this result (upper CI bound = 0.06) suggests potential statistical power limitations: prior studies reporting significant depression mediation typically employed larger samples (>500) with structural equation modeling, and post-hoc analysis suggests approximately 350–400 participants would be needed to detect an effect of this magnitude. Second, a population-specific mechanism may also contribute: in this peri-urban older adults sample exposed to chronic environmental stressors, the direct neurobiological impact of sleep disruption on cognition—such as impaired *β*-amyloid clearance via the glymphatic system (29)—may be sufficiently potent to overshadow the indirect affective pathway through depression. The attenuation of this indirect effect after controlling for lifestyle covariates (from −0.27 in the unadjusted model to −0.18) further suggests that confounding by health behaviors partly accounts for the observed sleep–depression–cognition association.

Given the low-income and peri-urban context of this population, we specifically recommend low-cost, scalable non-pharmacological interventions for sleep difficulties. Community-delivered Cognitive Behavioral Therapy for Insomnia (CBT-I), which has demonstrated robust efficacy (30) and can be administered in group formats by trained community health workers, represents the most evidence-based and cost-effective approach. Unlike hypnotic medications, CBT-I avoids polypharmacy risks and financial burden—concerns particularly salient for older adults already managing multiple chronic conditions (31). Standardized sleep hygiene education programs (regular sleep schedules, limited daytime napping, sleep-conducive environments) can be integrated into existing community health promotion activities at minimal additional cost.

In the sensitivity analysis excluding CDR ≥ 1 participants the PSQI coefficient maintained its negative direction but did not reach significance. This is likely attributable to two statistical factors rather than a substantive change in the sleep–cognition relationship: (1) the 19% reduction in sample size decreased statistical power, and (2) the exclusion of the most cognitively impaired participants restricted MoCA variance, attenuating detectable associations (32). The preserved coefficient direction suggests that sleep quality remains a relevant predictor across the cognitive spectrum, though detecting this effect requires sufficient outcome variance.

The current cross-sectional findings provide three specific foundations for future longitudinal research. First, by analyzing a cohort defined by strict local poverty criteria, this study establishes a socioeconomically homogeneous baseline (T₀) for tracking long-term poverty-related neurodegenerative trajectories. Second, the quantified effect sizes—particularly the PSQI predictive coefficient (*B* = −0.78) and the marginally significant ADL mediation trend (indirect effect = −0.19)—provide concrete parameters for sample size planning and causal hypothesis testing in future intervention trials. Third, the identification of sleep onset latency as the specific predictive PSQI component offers a precise, measurable target for longitudinal monitoring, enabling future studies to examine how temporal changes in sleep latency alter cognitive decline trajectories.

This study has the following limitations. Firstly, cross-sectional design cannot infer causal direction; Cognitive decline can also lead to difficulty falling asleep, reduced physical activity, and impaired ADL function, which requires longitudinal research to verify. Secondly, all measurements rely on self-report, and participants with cognitive impairment may have differential reporting biases regarding sleep quality. Thirdly, the sample comes from a single community, and its generalizability needs to be verified through research in multiple regions. Fourth, while the target community as a whole meets official low-income criteria (per capita income well below the 864 yuan/month Minimum Living Allowance standard per 2024 Tongliao municipal policy), income was not precisely quantified at the individual level. The characterization relies on community-level economic profiling and self-reported financial stress rather than individual tax or pension records, which may limit finer-grained socioeconomic stratification. Fifth, although the current analyses rigorously adjusted for smoking, alcohol consumption, and chronic disease count, other potentially important confounders such as physical activity levels, BMI nutritional status, and social support were not systematically measured.

## 5 Conclusion

This study advances our understanding of cognitive aging in resource-limited settings by elucidating the distinct factors associated with cognitive function among low-income older populations in China’s urban–rural fringe. We demonstrate that cognitive vulnerability in this demographic is strongly linked to the independent presence of physical frailty and specific sleep disturbances—notably prolonged sleep latency—alongside overarching chronic disease burdens, rather than being robustly explained by potential mediating factors like ADL impairment or depressive symptoms. By clarifying these associations, our findings highlight a clear, actionable public health directive: preserving cognitive health in this vulnerable group requires an integrated approach. Combining non-pharmacological, community-based sleep hygiene education with systematic chronic disease management represents a promising, highly targeted, and low-cost intervention framework that avoids the risks of polypharmacy.

## Data Availability

The raw data supporting the conclusions of this article will be made available by the authors, without undue reservation.
